# Supplementary material for: Pyruvate Dehydrogenase Kinase 1 inhibition mediated oxidative phosphorylation enhancement in cartilage promotes osteoarthritis progression
Source: BMC Musculoskelet Disord. 2023 Jul 20;24:597. doi: 10.1186/s12891-023-06585-6 (PMC10357736; doi:10.1186/s12891-023-06585-6)
Supplement: Supplementary file 1 — Additional file 1. [file 12891_2023_6585_MOESM1_ESM.zip › MF.Gsea.1653627316242/GOMF_VITAMIN_BINDING.html]

Details for gene set GOMF\_VITAMIN\_BINDING[GSEA]

|  || Dataset | 55457\_collapsed\_to\_symbols.cls.cls #OA\_versus\_normal.cls.cls #OA\_versus\_normal\_repos |
| Phenotype | cls.cls#OA\_versus\_normal\_repos |
| Upregulated in class | OA |
| GeneSet | GOMF\_VITAMIN\_BINDING |
| Enrichment Score (ES) | 0.32561368 |
| Normalized Enrichment Score (NES) | 1.5799093 |
| Nominal p-value | 0.0 |
| FDR q-value | 0.14612165 |
| FWER p-Value | 0.99 |
Table: GSEA Results Summary

  

Fig 1: Enrichment plot: GOMF\_VITAMIN\_BINDING      
 Profile of the Running ES Score & Positions of GeneSet Members on the Rank Ordered List

  

| SYMBOL | TITLE | RANK IN GENE LIST | RANK METRIC SCORE | RUNNING ES | CORE ENRICHMENT || 1 | FOLR1 | folate receptor alpha [Source:HGNC Symbol;Acc:HGNC:3791] | 8 | 1.202 | 0.0325 | Yes |
| 2 | LMBRD1 | LMBR1 domain containing 1 [Source:HGNC Symbol;Acc:HGNC:23038] | 40 | 1.009 | 0.0578 | Yes |
| 3 | GOT1 | glutamic-oxaloacetic transaminase 1 [Source:HGNC Symbol;Acc:HGNC:4432] | 59 | 0.974 | 0.0833 | Yes |
| 4 | S100G | S100 calcium binding protein G [Source:HGNC Symbol;Acc:HGNC:1436] | 92 | 0.925 | 0.1062 | Yes |
| 5 | P4HA2 | prolyl 4-hydroxylase subunit alpha 2 [Source:HGNC Symbol;Acc:HGNC:8547] | 258 | 0.779 | 0.1148 | Yes |
| 6 | ALAS1 | 5'-aminolevulinate synthase 1 [Source:HGNC Symbol;Acc:HGNC:396] | 288 | 0.757 | 0.1334 | Yes |
| 7 | UROS | uroporphyrinogen III synthase [Source:HGNC Symbol;Acc:HGNC:12592] | 323 | 0.735 | 0.1510 | Yes |
| 8 | GGCX | gamma-glutamyl carboxylase [Source:HGNC Symbol;Acc:HGNC:4247] | 331 | 0.734 | 0.1706 | Yes |
| 9 | TPK1 | thiamin pyrophosphokinase 1 [Source:HGNC Symbol;Acc:HGNC:17358] | 511 | 0.656 | 0.1747 | Yes |
| 10 | PLPBP | pyridoxal phosphate binding protein [Source:HGNC Symbol;Acc:HGNC:9457] | 637 | 0.610 | 0.1817 | Yes |
| 11 | MMACHC | metabolism of cobalamin associated C [Source:HGNC Symbol;Acc:HGNC:24525] | 642 | 0.608 | 0.1981 | Yes |
| 12 | IRX5 | iroquois homeobox 5 [Source:HGNC Symbol;Acc:HGNC:14361] | 733 | 0.585 | 0.2072 | Yes |
| 13 | P4HA1 | prolyl 4-hydroxylase subunit alpha 1 [Source:HGNC Symbol;Acc:HGNC:8546] | 833 | 0.560 | 0.2148 | Yes |
| 14 | ILVBL | ilvB acetolactate synthase like [Source:HGNC Symbol;Acc:HGNC:6041] | 898 | 0.543 | 0.2248 | Yes |
| 15 | OGFOD3 | 2-oxoglutarate and iron dependent oxygenase domain containing 3 [Source:HGNC Symbol;Acc:HGNC:26174] | 938 | 0.531 | 0.2364 | Yes |
| 16 | GCAT | glycine C-acetyltransferase [Source:HGNC Symbol;Acc:HGNC:4188] | 1095 | 0.497 | 0.2378 | Yes |
| 17 | LRAT | lecithin retinol acyltransferase [Source:HGNC Symbol;Acc:HGNC:6685] | 1205 | 0.475 | 0.2424 | Yes |
| 18 | GOT2 | glutamic-oxaloacetic transaminase 2 [Source:HGNC Symbol;Acc:HGNC:4433] | 1308 | 0.457 | 0.2470 | Yes |
| 19 | OGFOD1 | 2-oxoglutarate and iron dependent oxygenase domain containing 1 [Source:HGNC Symbol;Acc:HGNC:25585] | 1360 | 0.449 | 0.2554 | Yes |
| 20 | SHMT2 | serine hydroxymethyltransferase 2 [Source:HGNC Symbol;Acc:HGNC:10852] | 1518 | 0.426 | 0.2548 | Yes |
| 21 | NFS1 | NFS1 cysteine desulfurase [Source:HGNC Symbol;Acc:HGNC:15910] | 1741 | 0.393 | 0.2483 | Yes |
| 22 | PDXK | pyridoxal kinase [Source:HGNC Symbol;Acc:HGNC:8819] | 1923 | 0.370 | 0.2443 | Yes |
| 23 | PYGM | "glycogen phosphorylase, muscle associated [Source:HGNC Symbol;Acc:HGNC:9726]" | 1952 | 0.367 | 0.2522 | Yes |
| 24 | TAT | tyrosine aminotransferase [Source:HGNC Symbol;Acc:HGNC:11573] | 1979 | 0.363 | 0.2602 | Yes |
| 25 | CD320 | CD320 molecule [Source:HGNC Symbol;Acc:HGNC:16692] | 2044 | 0.357 | 0.2650 | Yes |
| 26 | OAT | ornithine aminotransferase [Source:HGNC Symbol;Acc:HGNC:8091] | 2074 | 0.353 | 0.2724 | Yes |
| 27 | CRABP1 | cellular retinoic acid binding protein 1 [Source:HGNC Symbol;Acc:HGNC:2338] | 2103 | 0.349 | 0.2799 | Yes |
| 28 | MTHFS | methenyltetrahydrofolate synthetase [Source:HGNC Symbol;Acc:HGNC:7437] | 2155 | 0.343 | 0.2853 | Yes |
| 29 | RBP1 | retinol binding protein 1 [Source:HGNC Symbol;Acc:HGNC:9919] | 2372 | 0.318 | 0.2772 | Yes |
| 30 | PHYH | phytanoyl-CoA 2-hydroxylase [Source:HGNC Symbol;Acc:HGNC:8940] | 2397 | 0.315 | 0.2840 | Yes |
| 31 | PYGB | glycogen phosphorylase B [Source:HGNC Symbol;Acc:HGNC:9723] | 2400 | 0.315 | 0.2925 | Yes |
| 32 | KYNU | kynureninase [Source:HGNC Symbol;Acc:HGNC:6469] | 2502 | 0.303 | 0.2929 | Yes |
| 33 | TKTL1 | transketolase like 1 [Source:HGNC Symbol;Acc:HGNC:11835] | 2528 | 0.300 | 0.2992 | Yes |
| 34 | GAD1 | glutamate decarboxylase 1 [Source:HGNC Symbol;Acc:HGNC:4092] | 2534 | 0.299 | 0.3071 | Yes |
| 35 | PC | pyruvate carboxylase [Source:HGNC Symbol;Acc:HGNC:8636] | 2629 | 0.287 | 0.3076 | Yes |
| 36 | FOLR2 | folate receptor beta [Source:HGNC Symbol;Acc:HGNC:3793] | 2670 | 0.283 | 0.3123 | Yes |
| 37 | P3H1 | prolyl 3-hydroxylase 1 [Source:HGNC Symbol;Acc:HGNC:19316] | 2853 | 0.265 | 0.3053 | Yes |
| 38 | SPTLC1 | serine palmitoyltransferase long chain base subunit 1 [Source:HGNC Symbol;Acc:HGNC:11277] | 2913 | 0.259 | 0.3079 | Yes |
| 39 | OPN3 | opsin 3 [Source:HGNC Symbol;Acc:HGNC:14007] | 2961 | 0.254 | 0.3112 | Yes |
| 40 | CRABP2 | cellular retinoic acid binding protein 2 [Source:HGNC Symbol;Acc:HGNC:2339] | 3058 | 0.244 | 0.3104 | Yes |
| 41 | FTCD | formimidoyltransferase cyclodeaminase [Source:HGNC Symbol;Acc:HGNC:3974] | 3064 | 0.244 | 0.3167 | Yes |
| 42 | DHTKD1 | dehydrogenase E1 and transketolase domain containing 1 [Source:HGNC Symbol;Acc:HGNC:23537] | 3112 | 0.239 | 0.3196 | Yes |
| 43 | PLOD1 | "procollagen-lysine,2-oxoglutarate 5-dioxygenase 1 [Source:HGNC Symbol;Acc:HGNC:9081]" | 3155 | 0.235 | 0.3228 | Yes |
| 44 | CBLIF | cobalamin binding intrinsic factor [Source:HGNC Symbol;Acc:HGNC:4268] | 3322 | 0.219 | 0.3158 | Yes |
| 45 | AFM | afamin [Source:HGNC Symbol;Acc:HGNC:316] | 3333 | 0.218 | 0.3210 | Yes |
| 46 | MMUT | methylmalonyl-CoA mutase [Source:HGNC Symbol;Acc:HGNC:7526] | 3362 | 0.215 | 0.3248 | Yes |
| 47 | SGPL1 | sphingosine-1-phosphate lyase 1 [Source:HGNC Symbol;Acc:HGNC:10817] | 3462 | 0.206 | 0.3227 | Yes |
| 48 | VDR | vitamin D receptor [Source:HGNC Symbol;Acc:HGNC:12679] | 3571 | 0.197 | 0.3197 | Yes |
| 49 | PLOD3 | "procollagen-lysine,2-oxoglutarate 5-dioxygenase 3 [Source:HGNC Symbol;Acc:HGNC:9083]" | 3614 | 0.192 | 0.3217 | Yes |
| 50 | OGFOD2 | 2-oxoglutarate and iron dependent oxygenase domain containing 2 [Source:HGNC Symbol;Acc:HGNC:25823] | 3632 | 0.191 | 0.3256 | Yes |
| 51 | PYGL | glycogen phosphorylase L [Source:HGNC Symbol;Acc:HGNC:9725] | 3826 | 0.174 | 0.3153 | No |
| 52 | GLDC | glycine decarboxylase [Source:HGNC Symbol;Acc:HGNC:4313] | 3913 | 0.163 | 0.3131 | No |
| 53 | KYAT3 | kynurenine aminotransferase 3 [Source:HGNC Symbol;Acc:HGNC:33238] | 4128 | 0.143 | 0.3003 | No |
| 54 | TYMS | thymidylate synthetase [Source:HGNC Symbol;Acc:HGNC:12441] | 4328 | 0.126 | 0.2882 | No |
| 55 | PAM | peptidylglycine alpha-amidating monooxygenase [Source:HGNC Symbol;Acc:HGNC:8596] | 4373 | 0.123 | 0.2881 | No |
| 56 | SRR | serine racemase [Source:HGNC Symbol;Acc:HGNC:14398] | 4397 | 0.121 | 0.2896 | No |
| 57 | SDS | serine dehydratase [Source:HGNC Symbol;Acc:HGNC:10691] | 4579 | 0.106 | 0.2784 | No |
| 58 | KL | klotho [Source:HGNC Symbol;Acc:HGNC:6344] | 4901 | 0.080 | 0.2555 | No |
| 59 | RBP4 | retinol binding protein 4 [Source:HGNC Symbol;Acc:HGNC:9922] | 5072 | 0.067 | 0.2440 | No |
| 60 | CBS | cystathionine beta-synthase [Source:HGNC Symbol;Acc:HGNC:1550] | 5285 | 0.049 | 0.2288 | No |
| 61 | SEC14L2 | SEC14 like lipid binding 2 [Source:HGNC Symbol;Acc:HGNC:10699] | 5435 | 0.038 | 0.2182 | No |
| 62 | DHFR | dihydrofolate reductase [Source:HGNC Symbol;Acc:HGNC:2861] | 5805 | 0.008 | 0.1895 | No |
| 63 | GC | GC vitamin D binding protein [Source:HGNC Symbol;Acc:HGNC:4187] | 5856 | 0.005 | 0.1857 | No |
| 64 | MTARC2 | mitochondrial amidoxime reducing component 2 [Source:HGNC Symbol;Acc:HGNC:26064] | 6076 | -0.010 | 0.1689 | No |
| 65 | DBH | dopamine beta-hydroxylase [Source:HGNC Symbol;Acc:HGNC:2689] | 6133 | -0.015 | 0.1649 | No |
| 66 | RLBP1 | retinaldehyde binding protein 1 [Source:HGNC Symbol;Acc:HGNC:10024] | 6239 | -0.023 | 0.1574 | No |
| 67 | EGLN1 | egl-9 family hypoxia inducible factor 1 [Source:HGNC Symbol;Acc:HGNC:1232] | 6364 | -0.032 | 0.1485 | No |
| 68 | RBP3 | retinol binding protein 3 [Source:HGNC Symbol;Acc:HGNC:9921] | 6380 | -0.034 | 0.1483 | No |
| 69 | THNSL2 | threonine synthase like 2 [Source:HGNC Symbol;Acc:HGNC:25602] | 6395 | -0.034 | 0.1482 | No |
| 70 | PSAT1 | phosphoserine aminotransferase 1 [Source:HGNC Symbol;Acc:HGNC:19129] | 6585 | -0.049 | 0.1347 | No |
| 71 | HDC | histidine decarboxylase [Source:HGNC Symbol;Acc:HGNC:4855] | 6599 | -0.050 | 0.1350 | No |
| 72 | PCCA | propionyl-CoA carboxylase subunit alpha [Source:HGNC Symbol;Acc:HGNC:8653] | 6627 | -0.052 | 0.1344 | No |
| 73 | HLCS | holocarboxylase synthetase [Source:HGNC Symbol;Acc:HGNC:4976] | 6759 | -0.060 | 0.1258 | No |
| 74 | TTPA | alpha tocopherol transfer protein [Source:HGNC Symbol;Acc:HGNC:12404] | 6796 | -0.063 | 0.1247 | No |
| 75 | ABAT | 4-aminobutyrate aminotransferase [Source:HGNC Symbol;Acc:HGNC:23] | 6804 | -0.063 | 0.1259 | No |
| 76 | ETNPPL | ethanolamine-phosphate phospho-lyase [Source:HGNC Symbol;Acc:HGNC:14404] | 6996 | -0.079 | 0.1131 | No |
| 77 | RHO | rhodopsin [Source:HGNC Symbol;Acc:HGNC:10012] | 7020 | -0.081 | 0.1136 | No |
| 78 | SCLY | selenocysteine lyase [Source:HGNC Symbol;Acc:HGNC:18161] | 7044 | -0.083 | 0.1140 | No |
| 79 | STRA6 | signaling receptor and transporter of retinol STRA6 [Source:HGNC Symbol;Acc:HGNC:30650] | 7107 | -0.089 | 0.1116 | No |
| 80 | PDXDC1 | pyridoxal dependent decarboxylase domain containing 1 [Source:HGNC Symbol;Acc:HGNC:28995] | 7373 | -0.108 | 0.0939 | No |
| 81 | MCCC1 | methylcrotonyl-CoA carboxylase subunit 1 [Source:HGNC Symbol;Acc:HGNC:6936] | 7401 | -0.111 | 0.0948 | No |
| 82 | MTARC1 | mitochondrial amidoxime reducing component 1 [Source:HGNC Symbol;Acc:HGNC:26189] | 7500 | -0.118 | 0.0904 | No |
| 83 | CALB1 | calbindin 1 [Source:HGNC Symbol;Acc:HGNC:1434] | 7543 | -0.122 | 0.0905 | No |
| 84 | TCN1 | transcobalamin 1 [Source:HGNC Symbol;Acc:HGNC:11652] | 7571 | -0.125 | 0.0918 | No |
| 85 | ALB | albumin [Source:HGNC Symbol;Acc:HGNC:399] | 7684 | -0.133 | 0.0867 | No |
| 86 | AGXT | alanine--glyoxylate and serine--pyruvate aminotransferase [Source:HGNC Symbol;Acc:HGNC:341] | 8115 | -0.164 | 0.0576 | No |
| 87 | FOLR3 | folate receptor gamma [Source:HGNC Symbol;Acc:HGNC:3795] | 8329 | -0.180 | 0.0459 | No |
| 88 | GPT | glutamic--pyruvic transaminase [Source:HGNC Symbol;Acc:HGNC:4552] | 8411 | -0.186 | 0.0447 | No |
| 89 | OGDH | oxoglutarate dehydrogenase [Source:HGNC Symbol;Acc:HGNC:8124] | 8503 | -0.194 | 0.0429 | No |
| 90 | DDC | dopa decarboxylase [Source:HGNC Symbol;Acc:HGNC:2719] | 8605 | -0.202 | 0.0405 | No |
| 91 | KYAT1 | kynurenine aminotransferase 1 [Source:HGNC Symbol;Acc:HGNC:1564] | 8633 | -0.203 | 0.0440 | No |
| 92 | MOCOS | molybdenum cofactor sulfurase [Source:HGNC Symbol;Acc:HGNC:18234] | 8721 | -0.210 | 0.0430 | No |
| 93 | C8G | complement C8 gamma chain [Source:HGNC Symbol;Acc:HGNC:1354] | 8871 | -0.223 | 0.0375 | No |
| 94 | ADH7 | "alcohol dehydrogenase 7 (class IV), mu or sigma polypeptide [Source:HGNC Symbol;Acc:HGNC:256]" | 8906 | -0.225 | 0.0410 | No |
| 95 | EGLN3 | egl-9 family hypoxia inducible factor 3 [Source:HGNC Symbol;Acc:HGNC:14661] | 8965 | -0.229 | 0.0428 | No |
| 96 | CYP2R1 | cytochrome P450 family 2 subfamily R member 1 [Source:HGNC Symbol;Acc:HGNC:20580] | 9182 | -0.246 | 0.0327 | No |
| 97 | GNMT | glycine N-methyltransferase [Source:HGNC Symbol;Acc:HGNC:4415] | 9208 | -0.248 | 0.0376 | No |
| 98 | MTR | 5-methyltetrahydrofolate-homocysteine methyltransferase [Source:HGNC Symbol;Acc:HGNC:7468] | 9213 | -0.249 | 0.0441 | No |
| 99 | GAD2 | glutamate decarboxylase 2 [Source:HGNC Symbol;Acc:HGNC:4093] | 9249 | -0.252 | 0.0483 | No |
| 100 | P3H2 | prolyl 3-hydroxylase 2 [Source:HGNC Symbol;Acc:HGNC:19317] | 9254 | -0.252 | 0.0549 | No |
| 101 | PANK3 | pantothenate kinase 3 [Source:HGNC Symbol;Acc:HGNC:19365] | 9286 | -0.255 | 0.0595 | No |
| 102 | SPTLC2 | serine palmitoyltransferase long chain base subunit 2 [Source:HGNC Symbol;Acc:HGNC:11278] | 9534 | -0.277 | 0.0478 | No |
| 103 | SHMT1 | serine hydroxymethyltransferase 1 [Source:HGNC Symbol;Acc:HGNC:10850] | 9727 | -0.294 | 0.0409 | No |
| 104 | CUBN | cubilin [Source:HGNC Symbol;Acc:HGNC:2548] | 9917 | -0.313 | 0.0347 | No |
| 105 | SLC19A1 | solute carrier family 19 member 1 [Source:HGNC Symbol;Acc:HGNC:10937] | 9931 | -0.315 | 0.0424 | No |
| 106 | PLOD2 | "procollagen-lysine,2-oxoglutarate 5-dioxygenase 2 [Source:HGNC Symbol;Acc:HGNC:9082]" | 10086 | -0.330 | 0.0394 | No |
| 107 | EGLN2 | egl-9 family hypoxia inducible factor 2 [Source:HGNC Symbol;Acc:HGNC:14660] | 10212 | -0.342 | 0.0391 | No |
| 108 | PNPO | pyridoxamine 5'-phosphate oxidase [Source:HGNC Symbol;Acc:HGNC:30260] | 10239 | -0.345 | 0.0465 | No |
| 109 | ALAS2 | 5'-aminolevulinate synthase 2 [Source:HGNC Symbol;Acc:HGNC:397] | 10247 | -0.346 | 0.0555 | No |
| 110 | ABCA4 | ATP binding cassette subfamily A member 4 [Source:HGNC Symbol;Acc:HGNC:34] | 10554 | -0.379 | 0.0420 | No |
| 111 | OGDHL | oxoglutarate dehydrogenase L [Source:HGNC Symbol;Acc:HGNC:25590] | 10754 | -0.399 | 0.0374 | No |
| 112 | TCN2 | transcobalamin 2 [Source:HGNC Symbol;Acc:HGNC:11653] | 10897 | -0.416 | 0.0378 | No |
| 113 | ACACB | acetyl-CoA carboxylase beta [Source:HGNC Symbol;Acc:HGNC:85] | 10955 | -0.425 | 0.0450 | No |
| 114 | CTH | cystathionine gamma-lyase [Source:HGNC Symbol;Acc:HGNC:2501] | 11108 | -0.444 | 0.0454 | No |
| 115 | SPTLC3 | serine palmitoyltransferase long chain base subunit 3 [Source:HGNC Symbol;Acc:HGNC:16253] | 11416 | -0.489 | 0.0348 | No |
| 116 | CYP2W1 | cytochrome P450 family 2 subfamily W member 1 [Source:HGNC Symbol;Acc:HGNC:20243] | 11635 | -0.523 | 0.0321 | No |
| 117 | CSAD | cysteine sulfinic acid decarboxylase [Source:HGNC Symbol;Acc:HGNC:18966] | 11649 | -0.525 | 0.0456 | No |
| 118 | ALDH1A2 | aldehyde dehydrogenase 1 family member A2 [Source:HGNC Symbol;Acc:HGNC:15472] | 11796 | -0.548 | 0.0492 | No |
| 119 | FASN | fatty acid synthase [Source:HGNC Symbol;Acc:HGNC:3594] | 12194 | -0.643 | 0.0359 | No |
| 120 | TKT | transketolase [Source:HGNC Symbol;Acc:HGNC:11834] | 12373 | -0.700 | 0.0412 | No |
Table: GSEA details [plain text format]

  

Fig 2: GOMF\_VITAMIN\_BINDING      
 Blue-Pink O' Gram in the Space of the Analyzed GeneSet

  

Fig 3: GOMF\_VITAMIN\_BINDING: Random ES distribution      
 Gene set null distribution of ES for **GOMF\_VITAMIN\_BINDING**
